# Analysis of Lifestyle‐Related Risk Factors in Stroke Patients

**DOI:** 10.1002/alz.090602

**Published:** 2025-01-09

**Authors:** Sanghun Nam, Young Myoung Lim, Ah‐Ram Kim, Seungju Lim, Ji‐Hyuk Park

**Affiliations:** ^1^ Yonsei New‐normal Lfiestyle Resarch Center, Yonsei University, Wonju, Gangwon‐do Korea, Republic of (South); ^2^ New Normal Lifestyle Research Institute / Yonsei University, Wonju, Gangwondo Korea, Republic of (South); ^3^ Yonsei University, Wonju, Gangwon‐do Korea, Republic of (South); ^4^ Yonsei Univsersity, Wonju Korea, Republic of (South)

## Abstract

**Background:**

Previous research has predominantly examined life satisfaction in stroke patients as a singular variable, overlooking its multidimensional nature encompassing health, income, spouse, and child satisfaction. To address this research gap, our longitudinal study utilizes survival analysis to investigate how various aspects of life satisfaction are influenced by diverse lifestyle factors.

**Method:**

We employed data from the Korean Longitudinal Study of Aging (2010‐2020), specifically focusing on stroke patients who survived from 2010 to 2020. Dependent variables included health, income, spouse, and child satisfaction. Lifestyle factors encompassed health behavior, health status, and social interactions, while Cox regression analysis incorporated control variables such as age, sex, education, and residential area.

**Result:**

Exercise demonstrated the highest hazard ratio (HR) in child satisfaction (HR = 2.111, CI = 1.04‐4.31), while health screening exhibited the highest HR in health satisfaction (HR = 1.489). Depression had the highest HR in child satisfaction (HR = 6.343, CI = 2.67‐14.98). The lowest HR in child satisfaction was observed for BMI (HR = 0.500, CI = 0.27‐0.94), whereas diabetes showed the highest HR (HR = 1.800, CI = 1.05‐3.08). Employment status had the highest HR in health satisfaction (HR = 1.678, CI = 1.68‐1.01), with no significant differences in other variables.

**Conclusion:**

Our study delves into the complex dynamics of the relationship between lifestyle factors and the various dimensions of life satisfaction among stroke patients. Through meticulous examination, we unravel the intricate connections that exist within this association.

